# Outer nuclear layer recovery as a predictor of visual prognosis in type 1 choroidal neovascularization of neovascular age-related macular degeneration

**DOI:** 10.1038/s41598-023-32184-5

**Published:** 2023-03-28

**Authors:** Seungheon Lee, Kyung Tae Kim, Dong Yoon Kim, Ju Byung Chae, Eoi Jong Seo

**Affiliations:** 1 Department of Ophthalmology, College of Medicine, Chungbuk National University Hospital, Chungbuk National University, 776, Sunhwan-1-Ro, Seowon-Gu, Cheongju, 28644 Korea; 2 Seoul Top Eye Center, Cheongju, Korea

**Keywords:** Eye manifestations, Prognostic markers

## Abstract

To investigate the changes in outer nuclear layer (ONL) thickness during anti-vascular endothelial growth factor (VEGF) treatment in type 1 choroidal neovascularization (CNV) and its impact on vision. Type 1 CNV eyes (n = 94) were retrospectively compared to normal control eyes (n = 35). Along with best-corrected visual acuity (BCVA), the location of CNV, foveal ONL thickness, and subretinal fluid height were measured using optical coherence tomography (OCT) and analyzed. Visual outcome and OCT biomarkers were compared. As a result, the CNV group had thinner foveal ONL and worse BCVA compared to the control group. ONL thickness recovered partially along with visual improvement following 3 monthly initial loading doses of aflibercept injections, and it correlated with the final BCVA during the 1-year follow-up. Eyes achieved foveal ONL recovery over + 10 µm had lower subfoveal CNV (45.5%) and showed better visual outcomes than eyes with stationary ONL or suboptimal ONL recovery (76.0%, *p* = 0.012). In conclusion, type 1 CNV eyes that recovered foveal ONL thickness at initial loading of anti-VEGF demonstrated good final visual outcome during the 1-year follow-up. Monitoring the foveal ONL thickness during early anti-VEGF treatment can give information about the visual outcomes in type 1 CNV.

## Introduction

Age-related macular degeneration (AMD) is a leading cause of blindness in the developed world; efforts have been made to prevent visual loss related to the disease^1,2^. Several anti-vascular endothelial growth factor (VEGF) agents with different treatment regimens have contributed to treating neovascular AMD (nAMD); however, the treatment outcome remains suboptimal for certain patients. Efforts have been made to identify the possible prognostic factors for visual outcome^3,4^.

Since the development of optical coherence tomography (OCT), the most significant OCT biomarker indicating photoreceptor function has been ellipsoid zone (EZ) integrity^5^. However, EZ can be influenced by various factors, such as image quality and media opacity. Furthermore, it can only be analyzed qualitatively. Recently, the thickness of the outer nuclear layer (ONL) has been considered as a quantitative OCT biomarker for photoreceptor function. Photoreceptor cell bodies lie within the ONL in a 7–8 folded sublayer structure^6^. Degeneration of the photoreceptor cell bodies can lead to thinning of the ONL, indicating that photoreceptor health can be estimated by measuring ONL thickness. Thinning of the ONL in chorioretinal disorders was observed not only in experimental animal models^7,8^ but also in humans^9–11^. ONL thinning in dry AMD is especially considered photoreceptor degeneration with future atrophy progression^12^.

However, little is known regarding the characteristics of ONL during treatment of diseases or its impact on visual prognosis, especially in nAMD. Therefore, we investigated whether ONL thickness can indicate photoreceptor function and visual prognosis when treating nAMD with an anti-VEGF agent, as in treating dry AMD. Investigating the changes in ONL thickness during treatment of nAMD and its relationship to other OCT parameters, such as subretinal fluid height and ellipsoid zone integrity, and knowledge in relation to visual prognosis can widen the understanding and perspective of the disease and its pathogenic mechanism.

## Methods

In this retrospective case–control study, we reviewed patients diagnosed with nAMD with type 1 choroidal neovascularization (CNV) at Chungbuk National University Hospital from January 2017 to June 2020. This study was approved by the Institutional Review Board of the Chungbuk National University Hospital, and it adhered to the tenets of the Declaration of Helsinki. The need for informed consent from the participants was waived by Institutional Review Board of the Chungbuk National University Hospital due to the retrospective nature of this study. For the diagnosis, all participants underwent an ophthalmic examination, including a best-corrected visual acuity (BCVA) examination, slit-lamp examination, fundus photography (TRC-NW8; Topcon Corporation, Tokyo, Japan), spectral domain (SD)-OCT (Spectralis OCT; Heidelberg Engineering, Heidelberg, Germany), fluorescein angiography (FA), and indocyanine angiography (ICGA). Fundus photography was taken under standard fundus photography with pupil dilation without steering. SD-OCT was taken with a standard volume scan, consisting of a 25-line horizontal raster scan covering 30° × 20° macula, centered on the fovea. FA and ICGA were taken simultaneously with a confocal scanning laser ophthalmoscope (Heidelberg Retina Angiography 2, HRA2; Heidelberg Engineering, Heidelberg, Germany) under standard protocol with consecutive serial image capture until 15 min after simultaneous intravenous injection of 3 ml of 10% sodium fluorescein and 2 ml of 12.5 mg/ml indocyanine green solution into one antecubital vein.

The inclusion criteria were as follows: (1) patients over 50 years of age with clinical features of nAMD; (2) patients diagnosed with type 1 CNV associated with subretinal fluid (SRF) and fibrovascular pigment epithelial detachment (PED) as per OCT^13^; (3) patients who completed the initial 3-month loading dose treatment with intravitreal anti-VEGF agent—aflibercept (Eylea, Regeneron, Armonk, NY, USA); (4) patients who had not received any previous therapy, including laser, photodynamic therapy, or anti-VEGF treatment; and (5) patients who had been followed-up for 1-year. The exclusion criteria were as follows: (1) patients diagnosed with type 2 or 3 CNV; (2) patients with secondary CNV from another cause, including pathologic myopia or chorioretinitis; (3) SRF from pachychoroid neovasculopathy or central serous chorioretinopathy; (4) patients with complete retinal layer thinning, which meets the criteria for complete retinal pigment epithelium (RPE) and outer retinal atrophy due to extensive retinal destruction^14^; (5) patients with comorbidities such as other retinal or choroidal disorder(s), including epiretinal membrane, vitreomacular traction, diabetic retinopathy, retinal vein occlusion, etc.; and (6) patients with poor quality of images, which precluded measurement of retinal layer thicknesses.

The CNV types were determined by the following criteria: type 1 CNVs were within the sub-RPE space, typically corresponding to angiographically occult CNV; type 2 CNVs were within the subretinal space, typically corresponding to angiographically classic CNV; and type 3 CNVs displayed intraretinal angiomatous proliferation^13^. All patients underwent multimodal imaging such as OCT, FA, and ICGA. If OCT was insufficient to determine the CNV type, then FA and ICGA were used as the reference. Intense hyperfluorescence that appeared early and showed progressive leakage was considered classic CNV and excluded. Staining/leakage with stippled hyperfluorescence and poorly demarcated areas of leakage in the late phase of FA was considered occult CNV and included. We only included type 1 CNVs in this study because ONL thickness measurement was impossible due to the disruption and destruction of the retinal laminar structure in the other CNV types. CNVs containing type 1 and type 2 lesions in a similar amount were classified as “type 2” and excluded. However, CNVs with predominantly type 1 characteristics with stippled leakage and staining pattern on FA without any retinal infiltrations were classified as type 1 and included. Type 1 CNVs accompanied by intraretinal fluid were also excluded because intraretinal layer thickness measurements are unclear in the presence of intraretinal fluid. Patients with thick choroid type 1 CNV such as aneurysmal type 1 CNV or polypoidal choroidal vasculopathy (PCV), which has a high prevalence in Asia, were included.

Overall, 94 eyes of 94 consecutive patients that met the inclusion criteria were enrolled in the study. Additionally, 35 normal eyes of 35 consecutive study participants who presented for mild cataracts or floaters and who did not have any morphological/functional retinal disorders were included as the control group. Based on fibrovascular PED identified by OCT, leakage and/or staining identified by FA, and the presence of CNV identified by ICGA, the relative location of the CNV to the foveal center was documented as subfoveal (CNV located under the foveal center), juxtafoveal (CNV located not under the foveal center but within the 1 mm foveal diameter circle), or extrafoveal (CNV located outside of the 1 mm foveal diameter circle)^15,16^. If a discrepancy existed among the three modalities, the CNV location was graded as the majority of modalities determined. The presence of posterior vitreous detachment (PVD) was also estimated with OCT and fundus photographs.

After a diagnosis of nAMD with type 1 CNV, the investigated patients were treated with an initial loading dose of aflibercept given via intravitreal injection every 3 months. After the loading period, patients were treated in bi- or trimonthly intervals as required by their clinical needs, following the treat & extend strategy^17,18^. Patients who had residual or increasing SRF with declining visual acuity on bimonthly aflibercept were switched to monthly ranibizumab (Lucentis; Genentech, San Francisco, CA, USA) or bevacizumab (Avastin; Genentech) at the ophthalmologist’s discretion. BCVA and foveal OCT data at baseline, after three loading doses of aflibercept, and at the 1-year follow-up were gathered and analyzed. The information regarding the total number of injections and follow-up duration was also documented.

The OCT biomarkers analyzed in the current study included: foveal ONL thickness, foveal SRF height, subfoveal choroidal thickness, and the presence of the foveal EZ. SRF height and subfoveal choroidal thickness were chosen because these biomarkers can be associated with the activity and vascularity of CNV. The data were produced with SD-OCT in the enhanced depth imaging mode. All OCT parameters were measured manually by using the computer-based caliper measurement tool in the SD-OCT system. On-axis images were selected and analyzed when several OCT images were available. An internal tracking mechanism, AutoRescan, was used to ensure that the repetitive scans were taken at the exact same location of the macula. The foveal ONL thickness was defined as the distance between the internal and external limiting membranes at the center of the fovea^19,20^. The foveal SRF height was measured from the tip of the RPE layer to the outer border of the detached retina at the fovea^21^. Subfoveal choroidal thickness was defined as the vertical distance from the outer border of the RPE to the inner border of the sclera at the fovea^22^. All of the aforementioned measurements were performed by two operators (EJS and SL) according to the same protocol without any knowledge of the other measurements. Intergrader agreement, measured with the intraclass correlation coefficient (ICC), was excellent between the two graders (see Supplementary Table S1). The mean of the two measurements was used in the analysis.

The presence of EZ was graded in a single horizontal scan through the fovea. EZ was defined as a hyperreflective line between the external limiting membrane and RPE, which is also hyperreflective in OCT. If the line was bold and distinct over 50% of the central fovea, the eye was considered as foveal EZ present, while if the line was weak and disrupted over 50% of the central fovea, the eye was regarded as foveal EZ absent^23–25^. The presence of EZ was graded at baseline, after three monthly loading doses of aflibercept, and at the 1-year follow-up. Each operator (EJS and SL) graded without the knowledge of the other’s grading. In case of a discrepancy between the two graders, a third blinded grader (KTK) adjudicated the decision. The inter-rater reliability was estimated with Cohen's kappa coefficient.

Baseline BCVA and OCT parameters were compared between the study and control groups. Changes in OCT parameters during treatment and their relationship to the final visual outcome were analyzed. The study group was subsequently divided into two subgroups, based on the difference in foveal ONL thickness at baseline and after three loading doses of aflibercept: the poor response group (group A) and the good response group (group B). The median change in foveal ONL thickness in the overall study population was used as the cut-off value, which was + 10 µm. The 10 µm cutoff was chosen because it is not only a median value of the ONL change in the current study, but also a value that can represent a mean ONL change after the treatment of chronic central serous chorioretinopathy^19,26,27^. Hence, the 94 eyes were categorized into 46 eyes for the poor responders (group A) and 48 eyes for the good responders (group B). BCVA and OCT parameters were compared between these groups.

SPSS version 21.0 (SPSS Inc, Chicago, IL, USA) was used for the statistical analysis. The independent t-test was used to compare variables between the study and control groups and between the subgroups. Chi-square analysis was used to compare EZ, age, and sex. Fisher’s exact test was used to compare the ratio of the CNV locations between subgroups. Multivariate linear regression was used to investigate the parameters that could predict the final visual outcome. In all the analyses, a value of *p* < 0.05 was considered as statistically significant.

## Results

Baseline characteristics of the type 1 CNV and control group patients are presented in Table 1. Type 1 CNV eyes had worse BCVA and a higher SRF height than the control eyes. Foveal ONL thickness measurements of the type 1 CNV group were also less than those of the control group. The foveal EZ was present in 36.2% of the study eyes. The inter-rater reliability between the two graders was fair (Cohen’s kappa coefficient was 0.397). Eyes with PVD had higher age than ones without PVD (72.6 years vs 64.2 years, *p* < 0.001).

**Table 1:** Baseline characteristics of type 1 CNV patients and the control group.

	Type 1 CNV (n: 94)	Control (n: 35)	*P* value
Sex (M/F)	65/29	17/18	0.040*
Age	68.9 ± 8.0	67.8 ± 6.5	0.501†
Refractive error (diopter)	+ 0.30 ± 1.37	− 0.10 ± 0.56	0.098†
BCVA (logMAR)	0.48 ± 0.28	0.04 ± 0.05	< 0.001†
PVD status (with/without/unidentified)	38/41/15	15/16/4	0.812*
CNV location (eyes)			
Subfovea (%)	58 (61.7)		
Juxtafovea (%)	30 (31.9)		
Parafovea (%)	6 (6.4)		
ONL thickness (μm)	54.0 ± 19.8	89.5 ± 10.3	< 0.001†
SRF height (μm)	174.4 ± 103.8	0	< 0.001†
Choroidal thickness (μm)	266.6 ± 100.8	276.3 ± 24.1	< 0.391†
Presence of foveal EZ	34 (36.2%)	35 (100%)	< 0.001*
Total anti-VEGF injections	7.1 ± 3.8	0	< 0.001†
Follow up duration (months)	12.2 ± 0.9	–	

*CNV* choroidal neovascularization, *BCVA* best-corrected visual acuity, *logMAR* log of minimum angle of resolution, *ONL* outer nuclear layer, *SRF* subretinal fluid, *VEGF* vascular endothelial growth factor, *PVD* posterior vitreous detachment.

*P value was calculated using the Chi-square analysis.

^†^*P* values were calculated using the Student’s *t* test.

### Foveal ONL change after anti-VEGF loading

**Figure 1:**
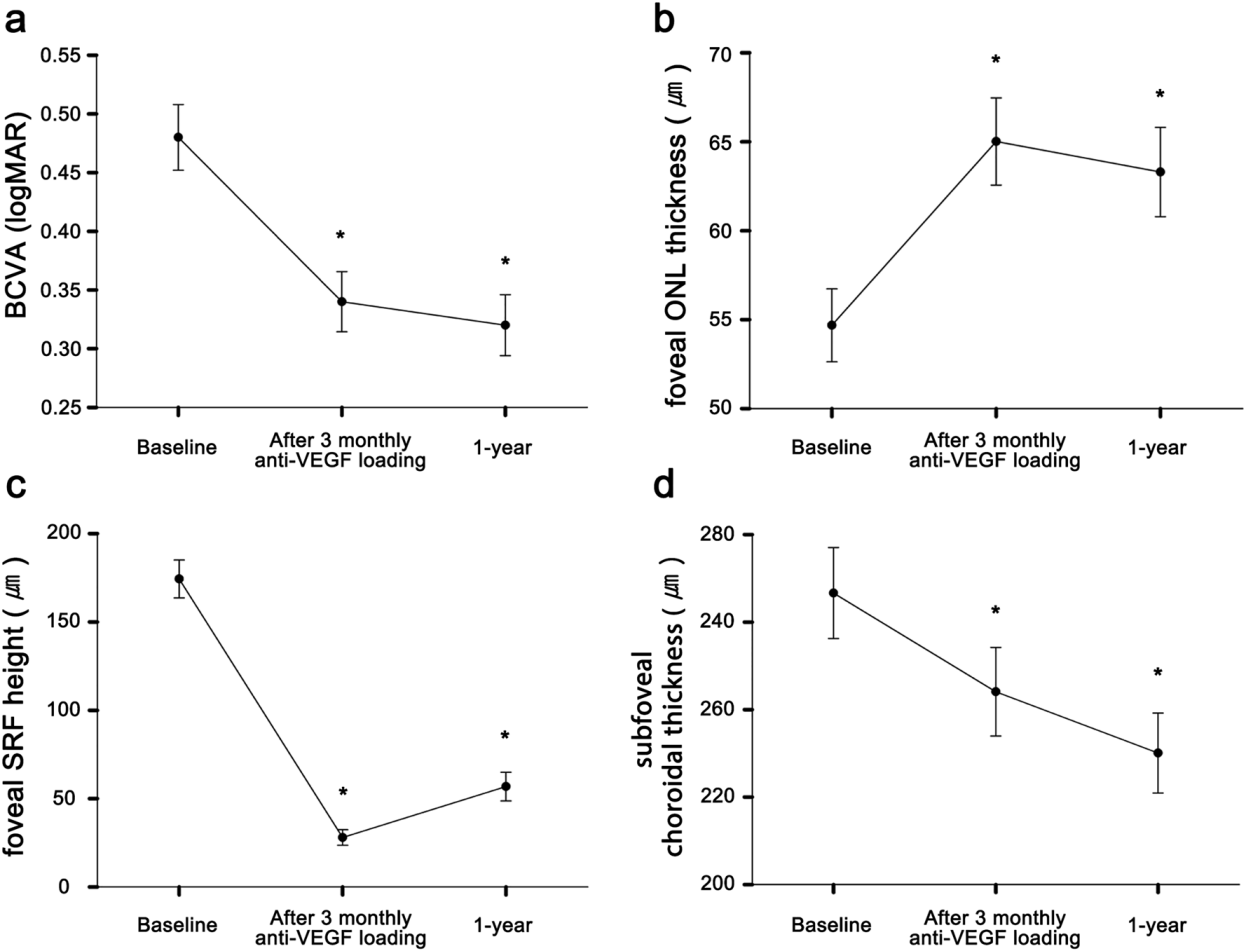
Changes in best-corrected visual acuity (BCVA) and optical coherence tomography (OCT) biomarkers during treatment in type 1 choroidal neovascularization. After intravitreal anti-vascular endothelial growth factor (VEGF) treatment, BCVA improved significantly (**a**). Simultaneously, a decreased foveal outer nuclear layer (ONL) thickness also recovered partially (**b**). The foveal subretinal fluid (SRF) height and subfoveal choroidal thickness also decreased (**c** and **d**). These improvements were maintained during the 12.2 ± 0.9-month follow-up period comprising 7.1 ± 3.8 intravitreal anti-VEGF injections. Asterisks indicate a significant difference from the baseline value.

As patients received their initial three intravitreal loading doses of aflibercept, both the SRF height and choroidal thickness decreased significantly compared to those at baseline (Fig. 1). Interestingly, the previously decreased ONL thickness also recovered partially alongside the treatment (54.68 ± 19.49 μm to 65.02 ± 23.84 μm, *p* < 0.001). With a morphological improvement, BCVA also improved compared to that at baseline (0.48 ± 0.28 to 0.34 ± 0.27). Improvements in the OCT biomarkers and BCVA were well-maintained until the 1-year follow-up period. During this period, the mean intravitreal anti-VEGF injection count was 7.1 ± 3.8.

### Factors affecting visual outcome before and after anti-VEGF loading

**Figure 2:**
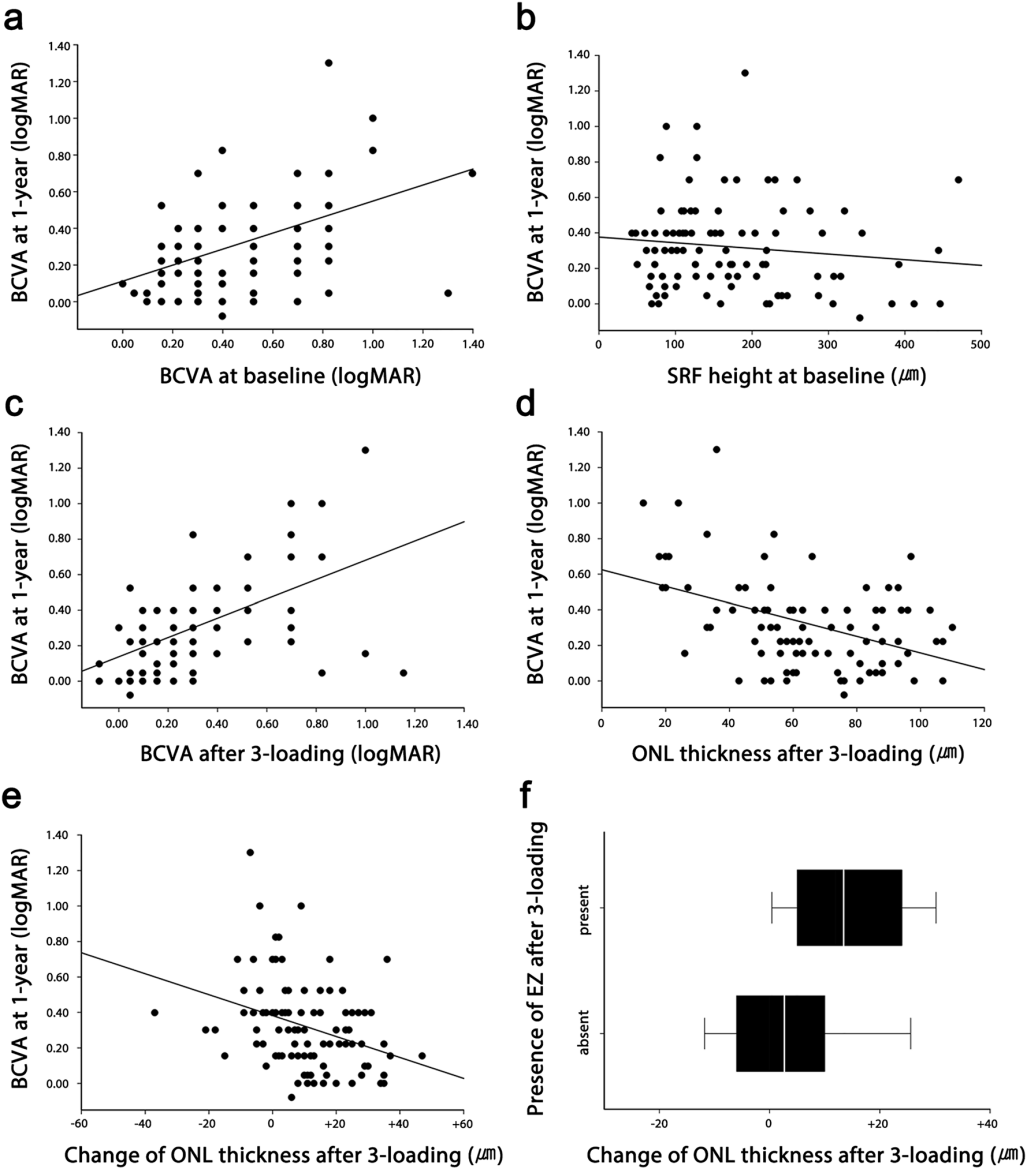
Analysis findings regarding the relationships among the optical coherence tomography parameters (OCT). Among the baseline OCT parameters, best-corrected visual acuity (BCVA) (*p* < 0.001) and foveal subretinal fluid (SRF) height (*p* = 0.004) are significantly correlated with the 1-year BCVA (**a** and **b**). Among OCT parameters after the three-monthly loading doses of aflibercept, the BCVA (*p* < 0.001) and foveal outer nuclear layer (ONL) thickness (*p* = 0.005) are significantly correlated with the 1-year BCVA (**c** and **d**). The ONL change between the baseline and after three monthly aflibercept loading doses also is significantly correlated with the 1-year BCVA (*p* = 0.001) (**e**). Eyes with a foveal ellipsoid zone (EZ) have a significantly greater recovery of the ONL than do eyes with an absent EZ (*p* = 0.001) (**f**).

Multivariate linear regression was performed to determine the prognostic factors for the visual outcome of 1-year follow-up among the early OCT biomarkers (Table 2). Among the baseline biomarkers of foveal ONL thickness, foveal SRF height, subfoveal choroidal thickness, and BCVA, we found that foveal SRF height and BCVA were significantly correlated with BCVA at the 1-year follow-up (Fig. 2A,B). A higher baseline SRF height was interestingly associated with a good 1-year BCVA. Baseline foveal ONL thickness and subfoveal choroidal thickness did not have a significant relationship with the 1-year BCVA. After three monthly loading doses of aflibercept, BCVA and foveal ONL thickness were significantly correlated with BCVA at the 1-year follow-up (Fig. 2C,D). Foveal SRF height and subfoveal choroidal thickness did not have a significant relationship with the 1-year BCVA. When analyzing the change in foveal ONL thickness before and after the three monthly aflibercept loading doses and the 1-year BCVA, the eyes with ONL improvement had a better 1-year BCVA, whereas the eyes with a stationary or worsening ONL had a poorer BCVA (Fig. 2E).

**Table 2:** Linear regression analysis of biomarkers in relation to the BCVA at 1-year follow-up.

Variables	Univariate analysis		Multivariate analysis	
	β	*P* value (95% CI)	β	*P* value (95% CI)
Baseline				
Duration of symptom	< 0.001	0.691 (− 0.001, 0.001)		
Foveal SRF height	− 0.001	0.015 (− 0.001, < − 0.001)	− 0.001	0.004 (− 0.001, < − 0.001)
Foveal ONL thickness	− 0.002	0.070 (− 0.005, < 0.001)		
Foveal choroidal thickness	< 0.001	0.329 (− 0.001, < 0.001)		
BCVA	0.469	< 0.001 (0.295, 0.642)	0.511	< 0.001 (0.342, 0.680)
After 3 aflibercept loading				
Foveal SRF height	0.001	0.142 (< 0.001, 0.002)		
Foveal ONL thickness	− 0.003	0.002 (− 0.005, − 0.001)	− 0.003	0.002 (− 0.005, − 0.001)
Foveal choroidal thickness	< 0.001	0.210 (− 0.001, < 0.001)		
BCVA	0.409	< 0.001 (0.227, 0.591)	0.444	< 0.001 (0.265, 0.623)

*SRF* subretinal fluid, *ONL* outer nuclear layer, *BCVA* best corrected visual acuity, *β* regression coefficient, *CI* confidence interval.

The presence of the foveal EZ also increased after three monthly loading doses of aflibercept from 34 (36.2%) eyes to 68 (72.3%) eyes. Inter-rater reliability was substantial (Cohen’s kappa coefficient was 0.625). Eyes with a present foveal EZ had a significantly greater increase in the ONL after three monthly loading doses of aflibercept (*p* = 0.001). The presence of a foveal EZ in the eyes also had a significant relationship with the 1-year BCVA. At the 1-year follow-up, eyes with a present foveal EZ had a mean BCVA of 0.26 ± 0.22, whereas eyes with an absent foveal EZ had a mean BCVA of 0.48 ± 0.27 (*p* < 0.001). PVD status did not affect BCVA, ONL thickness, SRF height, choroidal thickness, and their changes over one year.

### Subgroup analysis based on foveal ONL improvement

**Figure 3:**
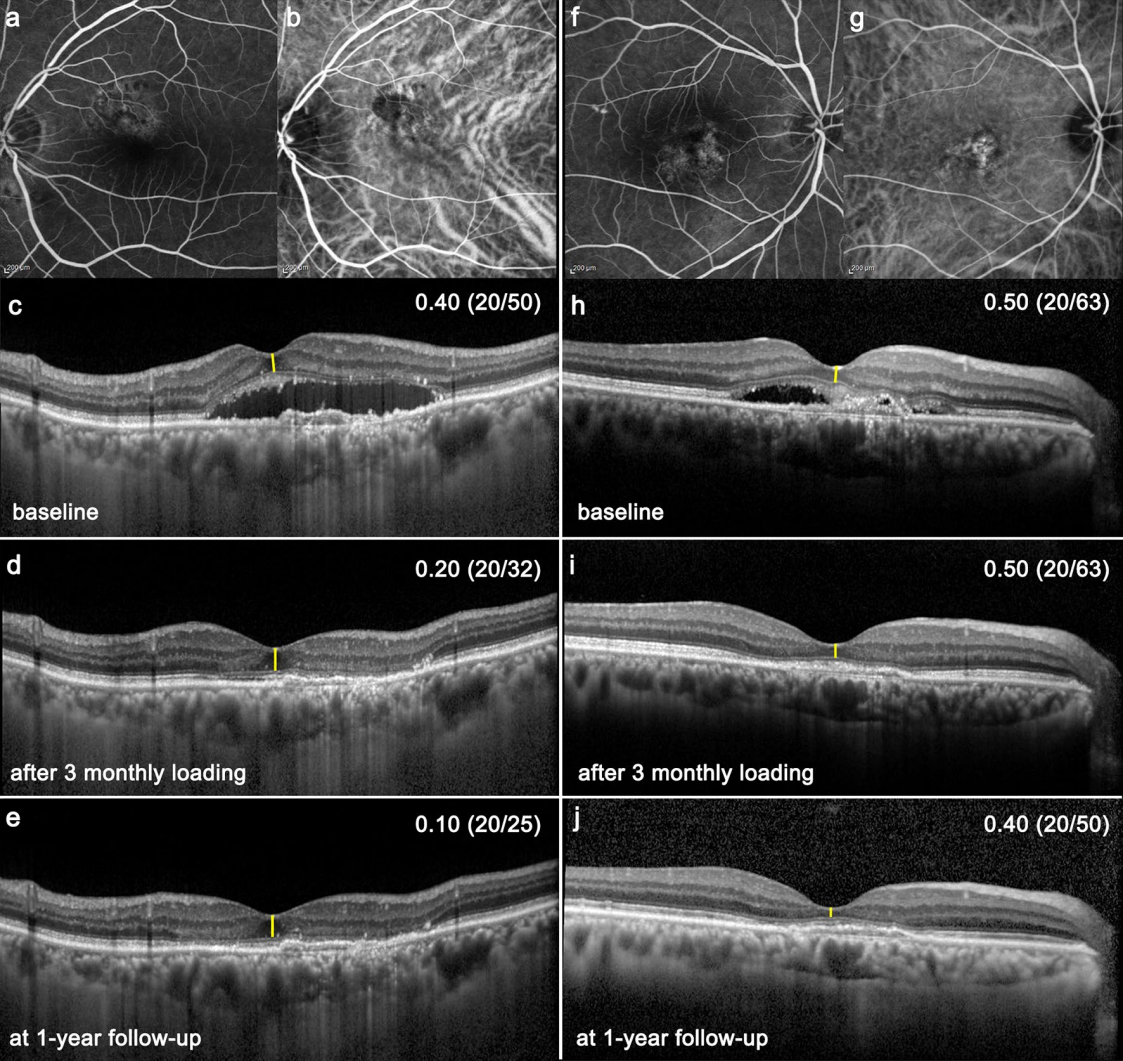
Representative images of the subgroup A and B. The left column images (**a**, **b**, **c**, **d**, and **e**) represent group B (i.e., good response), while the right column images (**f**, **g**, **h**, **i**, and **j**) represent group A (i.e., poor response). Best-corrected visual acuity (BCVA), using the log of minimum angle of resolution and Snellen (parenthesis), is documented on the right upper corner of each image. At baseline, type 1 choroidal neovascularization** (**CNV) is diagnosed with fluorescein angiography (**a** and **f**), indocyanine green angiography (**b** and **g**), and optical coherence tomography (**c** and **h**). The group B eye has juxtafoveal CNV while the group A eye has subfoveal CNV. Both eyes have similar ONL thicknesses (**c** and **h**, 59 µm and 45 µm, respectively) and vision at baseline. After 3 initial monthly loading doses with aflibercept, the subretinal fluid is well resorbed in both the groups; however, the ONL thickness and BCVA recovered in the group B eyes (**d**, 88 µm), while they remained unchanged in the group A eyes (**i**, 45 µm). These differences were maintained until the 1-year follow-up (**e** and **j**, 88 µm, and 40 µm, respectively). The yellow lines indicate the foveal ONL thickness.

Subsequently, the study eyes were divided into two groups, based on their foveal ONL thickness response to the initial three aflibercept loading doses: the poor response group (group A) and the good response group (group B) (Table 3). Group B showed greater recovery of ONL thickness and improvement in the BCVA than did group A. Of interest, group A had more eyes with subfoveal CNV than did group B (76.0% vs 45.5%, *p* = 0.012). Representative cases from each group are presented in Fig. 3.

**Table 3:** A comparison between group A and group B.

	Group A (n = 46)	Group B (n = 48)	*P* value
ONL change (µm)‡	− 1.3 ± 8.9	+ 21.5 ± 9.0	< 0.001*
Number of injections	7.3 ± 3.8	6.9 ± 3.7	0.621*
Duration of symptom (days)	36.5 ± 42.8	34.5 ± 93.6	0.903*
PVD status (with/without)	20/23	18/18	0.823
BCVA (logMAR)			
Baseline	0.51 ± 0.24	0.45 ± 0.31	0.250*
After 3 loading doses	0.41 ± 0.24	0.27 ± 0.25	0.010*
At 1-year	0.43 ± 0.27	0.22 ± 0.19	< 0.001*
ONL thickness (μm)			
Baseline	53.0 ± 22.7	56.3 ± 15.9	0.404*
After 3 loading doses	51.6 ± 21.8	77.9 ± 18.1	< 0.001*
At 1-year	56.7 ± 26.8	69.6 ± 20.0	0.010*
SRF height (μm)			
Baseline	165.6 ± 84.0	178.2 ± 110.7	0.350*
After 3 loading doses	37.3 ± 44.8	19.0 ± 38.9	0.046*
At 1-year	64.1 ± 89.9	49.8 ± 65.9	0.377*
CNV location			0.012†
Subfovea	35 (76.0%)	23 (45.5%)	
Juxtafovea (< 1 mm diameter circle)	10 (20.0%)	20 (45.5%)	
Parafovea (> 1 mm diameter circle)	1 (4.0%)	5 (9.0%)	

*ONL* outer nuclear layer, *SRF* subretinal fluid, *CNV* choroidal neovascularization, *BCVA* best-corrected visual acuity, *logMAR* log of minimum angle of resolution.

*P values were calculated using the Student’s *t* test.

^†^*P* value was calculated using the Fisher’s exact test.

^‡^ONL change between the baseline and after 3 initial loading doses.

## Discussion

In the present study, we observed that patients with type 1 CNV had a thinner foveal ONL thickness compared to the healthy controls; the reduced foveal ONL thickness partially recovered with anti-VEGF treatment, with a corresponding improvement in BCVA; and an increased ONL thickness after the three initial loading doses of aflibercept was related to a good visual outcome in type 1 CNV associated with nAMD during the 1-year observation period.

Foveal ONL thinning is observed not only in various chorioretinal disorders^10,28^ but also in relatively benign conditions such as drusen or pachychoroid pigment epitheliopathy^9,11^, indicating that ONL may correspond very sensitively to photoreceptor vitality. Thick ONL is considered a predictor for SRF resorption in chronic central serous chorioretinopathy treated with photodynamic therapy^29^. In this study, nAMD eyes also showed more ONL thinning and decreased visual acuity on baseline testing compared to the control eyes. Interestingly, during the initial loading doses, the foveal ONL thickness recovered partially as vision improved simultaneously. This phenomenon can be explained by the hypothesis of “a point of no return.” Photoreceptor cell bodies that have degenerated and crossed the point of no return are unable to recover their functional capabilities despite treatment. However, cell bodies with decreased function that have not yet reached the apoptosis stage could be salvaged.

Therefore, the amount of salvageable photoreceptor cell bodies could be considered as a prognostic factor for good visual outcome. By measuring the amount of ONL that was restored, we assumed that the eyes with ONL improvement had more photoreceptor cell bodies that could be salvaged after the loading doses were administered compared to the eyes without ONL improvement. As a result, ONL thickness after three monthly loading doses and the change in the ONL thickness during the loading doses had a significant positive correlation with the 1-year visual outcome.

Why then, do certain eyes have many salvageable photoreceptors and good visual prognosis? Several studies have demonstrated that poor visual prognosis is associated with the presence of subfoveal vascularized PED in nAMD, or even in central serous chorioretinopathy^30–32^. In the current study, we found that group A had more eyes with subfoveal CNV than did group B. Since we included only type 1 CNV cases, direct infiltration stretching from CNV into the retinal tissue was minimal. Even if CNV did not penetrate the RPE tight junction barrier, it could still harm the photoreceptor vitality and vitiate vision when it lay subfoveally.

The integrity of the EZ band has a strong correlation with visual acuity/function^5^. In this study, foveal EZ presence after three loading doses was also associated with good ONL thickness recovery and 1-year visual outcome. However, the low inter-rater reliability at baseline indicated that a discrepancy can exist between graders for judging the presence of foveal EZ, especially when SRF exists. Inter-rater reliability increased as SRF decreased with aflibercept loading doses. Measuring the thickness of ONL can provide consistent information about visual function, regardless of the presence of SRF, with an excellent intergrader agreement.

An intriguing point of our results is that the baseline foveal SRF height had a positive correlation with the 1-year BCVA (Fig. 2B). Many OCT biomarkers, including the SRF, intraretinal fluid, PED, sub-RPE fluid, and subretinal hyperreflective material were investigated for their influence on visual outcome^33,34^. Among them, the significance of the presence of SRF remains controversial. Some studies found that the SRF was a negative predictor for BCVA in nAMD^35,36^, while others reported that SRF was associated with a good visual outcome^37,38^. In our study, the baseline SRF height was correlated with the 1-year BCVA, but it was not correlated with ONL thickness or EZ integrity. An increased baseline SRF height can be correlated with a good 1-year BCVA, although the impact of SRF on photoreceptor vitality is inconclusive.

Choroidal thickness is generally decreased in eyes with nAMD, compared to its thickness in normal eyes. However, the baseline choroidal thickness in our study group was similar to that in the control group. An explanation for this finding is that eyes with thick choroid type-1 CNV, such as PCV, were included in the study group. Typical nAMD and PCV showed choroidal thickness differences at baseline, but no difference existed in the study results such as a change in BCVA and ONL thickness (see Supplementary Fig. S1 and Table S2). Therefore, we set typical nAMD and PCV as a single study group and interpreted the results. We also observed choroid thinning after anti-VEGF treatment, as in other reports^39,40^. Future studies on the influence of anti-VEGF drugs on using OCT angiography or measuring choroidal vascularity index could aid the understanding of the treatment mechanism. PVD status also did not affect the ONL thickness and its change over 1-year. Three eyes showed vitreomacular adhesion (VMA) in eyes without PVD but did not show any morphological change due to traction. We consider that the influence of VMA on the study result is minimal because the number of eyes is too small, and PVD status did not affect the ONL change. Future studies with a more significant number of VMA can clarify the impact of VMA on ONL changes.

Our present study was a single-center, retrospective case–control study and had some limitations. An important limitation was that all measurements were conducted manually rather than via an automated method. To avoid bias, each measurement was performed separately by two ophthalmologists and averaged. The intergrader agreement was excellent. The measurement error, which can be related to off-axis measurements, was minimized because we selected the on-axis images as much as possible, and we made measurements by using the computer-based caliper in the SD-OCT system rather than by extracting measurements from the image. An SD-OCT system can compensate for the vertical/horizontal scale difference. Next, a 10 μm threshold cut-off, which was the median value of ONL thickness change after aflibercept loading, was used as the criterion to categorize the study eyes into subgroups. On account that the categorization of a continuous variable by using a threshold cut-off may cause biased results, we initially reported the findings of continuous variables, based on regression analysis. We then strengthened the findings by using categorization analysis. We also chose the cut-off value based on the mean ONL change from other reports^19,26,27^. Finally, BCVA was the only method for measuring the function of the foveal photoreceptors. Objective methods of examining photoreceptor functions, including multifocal electroretinography, retinal microperimetry, or adaptive optics, could provide further information about photoreceptor function rather than subjective tests of visual acuity.

In summary, the foveal ONL thickness may indicate foveal photoreceptor vitality. Improvements in ONL thickness during the initial three loading doses with intravitreal anti-VEGF were considered to indicate photoreceptor viability versus irreversible cell death and hence, correlated with a good 1-year visual prognosis. Early improvement in the foveal ONL thickness with anti-VEGF treatment may be advantageous as a clinical indicator for the remaining healthy photoreceptors and may be a positive prognostic factor for visual acuity in type 1 CNV from nAMD.

## Supplementary Information

Supplementary Figure S1.

Supplementary Table S1.

Supplementary Table S2.

## Data Availability

The datasets generated during and/or analyzed during the current study are available from the corresponding author on reasonable request.
