# Supplementary material for: Outer nuclear layer recovery as a predictor of visual prognosis in type 1 choroidal neovascularization of neovascular age-related macular degeneration
Source: Sci Rep. 2023 Mar 28;13:5045. doi: 10.1038/s41598-023-32184-5 (PMC10050211; doi:10.1038/s41598-023-32184-5)
Supplement: Supplementary file 1 — Supplementary Figure S1. [file 41598_2023_32184_MOESM1_ESM.pdf]

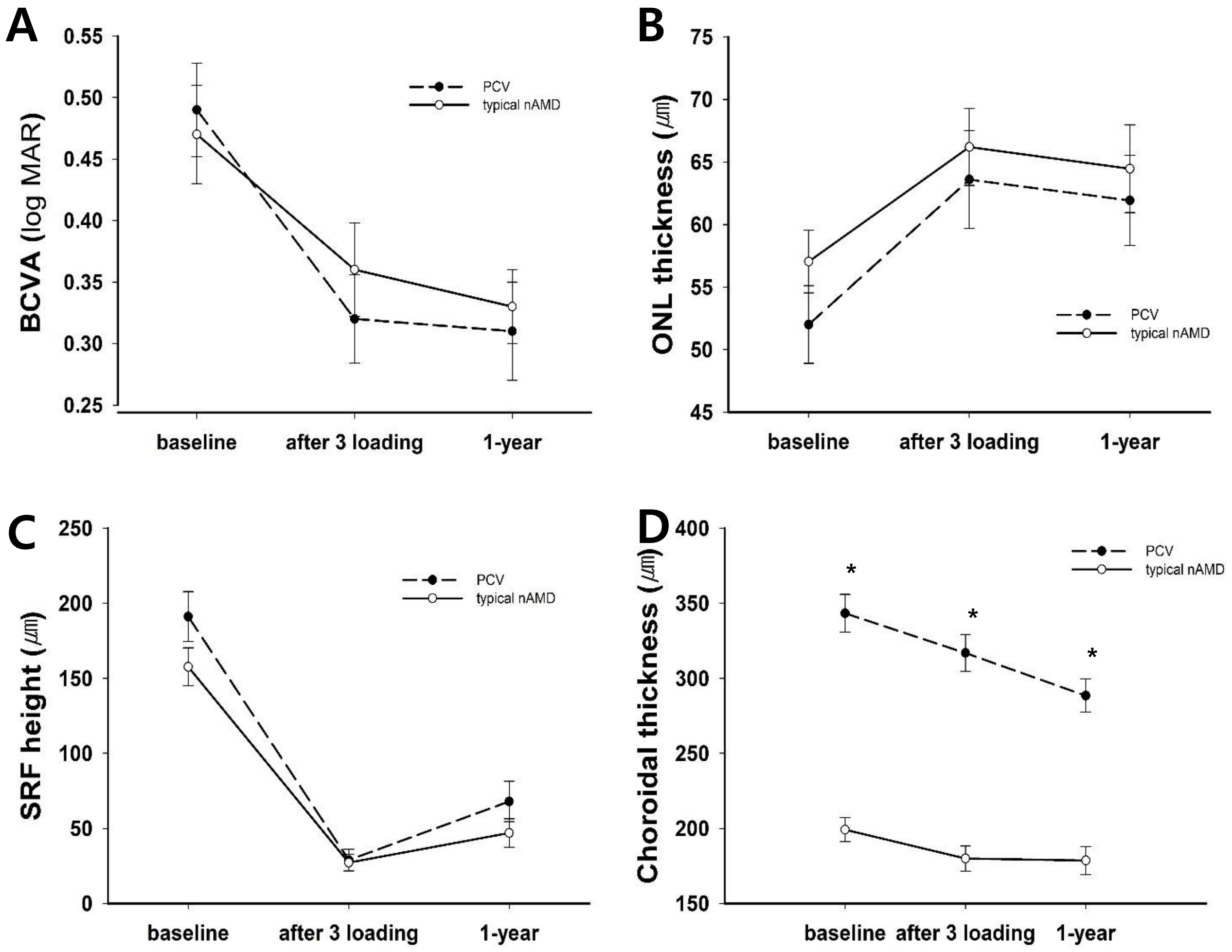

Supplementary Fig. S1 Comparison between polypoidal choroidal vasculopathy (PCV) and typical neovascular age-related macular degeneration (nAMD).

PCV and typical nAMD showed no difference in best-corrected visual acuity (BCVA), outer nuclear layer (ONL) thickness change, and subretinal fluid (SRF) height in 1-year follow-up (A, B, and C). However, PCV showed significantly increased choroidal thickness compared to typical nAMD throughout the whole study period (D).
