# Supplementary material for: Outer nuclear layer recovery as a predictor of visual prognosis in type 1 choroidal neovascularization of neovascular age-related macular degeneration
Source: Sci Rep. 2023 Mar 28;13:5045. doi: 10.1038/s41598-023-32184-5 (PMC10050211; doi:10.1038/s41598-023-32184-5)
Supplement: Supplementary file 2 — Supplementary Table S1. [file 41598_2023_32184_MOESM2_ESM.pdf]

Supplementary Table S1. Intergrader agreement of optical coherence tomography parameters between the two graders.

	Intra-class correlation	95% confidence interval
**Baseline ONL**	0.995	0.992,0.996
**Baseline SRF**	0.999	0.999,1.000
**Baseline choroidal thickness**	0.982	0.974,0.987
**After loading ONL**	0.994	0.991,0.996
**After loading SRF**	0.994	0.991,0.996
**After loading choroidal thickness**	0.997	0.995,0.998
**1-year ONL**	0.978	0.968,0.985
**1-year SRF**	0.998	0.997,0.998
**1-year choroidal thickness**	0.973	0.962,0.982

ONL, outer nuclear layer; SRF, subretinal fluid;
