# Supplementary material for: Outer nuclear layer recovery as a predictor of visual prognosis in type 1 choroidal neovascularization of neovascular age-related macular degeneration
Source: Sci Rep. 2023 Mar 28;13:5045. doi: 10.1038/s41598-023-32184-5 (PMC10050211; doi:10.1038/s41598-023-32184-5)
Supplement: Supplementary file 3 — Supplementary Table S2. [file 41598_2023_32184_MOESM3_ESM.pdf]

Supplementary Table S2. Comparison between polypoidal choroidal vasculopathy (PCV) and typical neovascular age-related macular degeneration (nAMD)

	Typical type 1 nAMD (n: 50)	PCV (n: 44)	P value
**Sex (M/F)**	33/17	32/12	0.481
**Age**	71.2±6.4	66.2±8.9	0.002
**Refractive error (diopter)**	+0.36±1.37	+0.24±1.39	0.685
**Total anti-VEGF injections**	6.8±3.7	7.3±3.8	0.505
**BCVA (logMAR)**			
**baseline**	0.47±0.29	0.49±0.25	0.671
**after 3 loading doses**	0.36±0.26	0.31±0.24	0.333
**at 1-year**	0.33±0.27	0.31±0.25	0.607
**ONL thickness (μm)**			
**baseline**	52.0±20.6	57.0±18.3	0.213
**after 3 loading doses**	63.6±26.2	66.3±21.7	0.594
**at 1-year**	61.9±24.0	64.5±24.8	0.618
**SRF height (μm)**			
**Baseline**	157.7±88.9	191.1±109.9	0.107
**after 3 loading doses**	27.2±38.5	28.9±47.4	0.850
**at 1-year**	46.9±67.0	67.9±89.2	0.197
**Choroidal thickness (μm)**			
**Baseline**	199.1±56.9	343.4±83.4	<0.001
**after 3 loading doses**	179.9±59.1	316.9±82.1	<0.001
**at 1-year**	178.6±66.7	288.5±73.3	<0.001

ONL, outer nuclear layer; SRF, subretinal fluid; BCVA, best-corrected visual acuity; logMAR, log of minimum angle of resolution
